# High Prevalence of Human Papillomavirus Infection among Brazilian Pregnant Women with and without Human Immunodeficiency Virus Type 1

**DOI:** 10.1155/2009/485423

**Published:** 2009-09-10

**Authors:** Emilia Moreira Jalil, Geraldo Duarte, Patrícia El Beitune, Renata Toscano Simões, Patrícia Pereira dos Santos Melli, Silvana Maria Quintana

**Affiliations:** ^1^STD Unit, Brazilian Program of STD/Aids, Secretariat for Health Surveillance, Ministry of Health, 70070-600 Brasília, DF, Brazil; ^2^Department of Gynecology and Obstetrics, Faculty of Medicine of Ribeirão Preto, University of São Paulo, 14049-900 Ribeirão Preto, SP, Brazil; ^3^Department of Obstetrics and Gynecology, Medical School of the Federal University of Health Sciences of Porto Alegre, 90020-090 Porto Alegre, RS, Brazil; ^4^Department of Medicine, Faculty of Medicine of Ribeirão Preto, University of São Paulo, 30150-221 Ribeirão Preto, SP, Brazil

## Abstract

*Objective*. To estimate HPV prevalence among pregnant women from Ribeirão Preto, Brazil, and the possible influence of HIV-1 infection on this prevalence. *Methods*. A cross-sectional study with 44 HIV-positive and 53 HIV-negative pregnant women was conducted. Cervicovaginal specimens were obtained from all women during gynecologic exam. HPV DNA, low and high risk HPV types, was detected using conventional PCR. Statistical analysis used Student's *t*-test, Mann-Whitney test, Fischer's Exact test, and prevalence ratios with 95% confidence interval. *Results*. HIV-positive pregnant women had higher proportion of HPV infection than HIV-negative pregnant women (79.5% versus 58.5%; *P* < .05). HPV positivity prevalence ratio for HIV-positive women was 1.36 (95% CI 1.04–1.8; *P* = .03). There was significant association between HIV viral load levels and HPV positivity (*P* < .05). *Conclusions*. Our results demonstrate higher HPV positivity in HIV-infected pregnant women. Higher values of HIV viral load were associated with HPV positivity.

## 1. Introduction

The human papillomavirus (HPV) infection is the most common sexually transmitted disease (STD) worldwide, representing a significant health problem due to its high prevalence and transmissibility. It is estimated that 75 percent of the sexually active population has been exposed to HPV [1]. Prevalence estimates vary according to the diagnostic method and the population examined, with higher rates being observed in studies using molecular biology and including young women with high-risk sexual behavior [2].

Women living with human immunodeficiency virus (HIV) are particularly susceptible to HPV infection with elevated prevalence rates [3] and higher HPV positivity when compared with women without HIV infection [4]. They also present more commonly persistent infections and cervical intraepithelial lesions [5]. The HPV positivity depends on plasma HIV viral load and CD4 T cell count, mainly below 200 cells/mm^3^ [6, 7].

It was hypothesized that pregnancy might interfere with HPV infection due to physiological immune modifications [8]. The most feared consequence of HPV infection during pregnancy is the occurrence of juvenile laryngeal papillomatosis, which is rare but is associated with significant morbidity and linked to mother-child transmission of the virus [9]. HPV prevalence rates vary widely in pregnant women, ranging from 5.5% to 65% [10].

Few studies have evaluated the interaction between pregnancy and HIV/HPV coinfection. Bolen et al. [11] identified an overall HPV prevalence of 35.5% among HIV-positive pregnant Thai women. Similar rates were found in a North-American cohort of HIV-infected pregnant women [12]. Taking into account the scarce data available about this theme and the relevance of HPV infection for public health, the objective of the present study was to estimate HPV prevalence among pregnant women from Ribeirão Preto, Brazil, and to identify the possible influence of HIV-1 infection on this prevalence.

## 2. Materials and Methods

A cross-sectional study was conducted from May 2006 to February 2007 on 44 HIV-positive pregnant women from the Prenatal Outpatient Clinic of the Infectious Diseases Unit and on 53 HIV-negative pregnant women from the Low-Risk Prenatal Outpatient Clinic of the Obstetrics and Gynecology Department of the University Hospital, Medical School of Ribeirão Preto, University of São Paulo. This service is a tertiary hospital with a large catchment population mainly of middle and low socioeconomic status. Women aged 15–40 years and with more than 21 weeks of gestation were selected for both groups. HIV-infected pregnant women had a previous or current diagnosis of the infection and no other disease, whereas non-HIV-infected women had no diseases and were considered normal from a clinical and serologic viewpoint.

The study protocol was approved by the Ethics Review Board of the hospital, and all women gave written informed consent to participate. A structured questionnaire was applied by the researchers in a personal interview to obtain sociodemographic characteristics, medical, reproductive, contraceptive, and sexual history, and laboratory data. All women had a gynecologic examination that included a Papanicolaou smear and the collection of cervicovaginal specimens which were stored in 5 mL of sterile saline solution on the same day at −80° until processing. DNA extraction was performed as previously described [13]. We included both low and high risk HPV types.

The extracted preparations were assessed by the polymerase chain reaction (PCR) using *β*-globin primers PCO3 and PCO4 [14] to confirm the presence of an adequate quality of DNA and the absence of inhibitors. HPV DNA was detected by conventional PCR with consensus primers GP5+/GP6+, which amplify a 150 base pair (bp) fragment from the L1 open reading frame of a broad spectrum of mucosotropic HPV types [15]. PCR was carried out in a 25 *μ*L reaction mix containing 5% glycerol, 3.0 *μ*M MgCl_2_, 20 mM of each deoxynucleoside triphosphate, 0.6 mM of each primer (Fermentas, Canada), 1× buffer (200 mM Tris-HCl, pH 8.4, and 500 mM KCl), 1.25 units of DNA polymerase (Invitrogen, USA), and 1 *μ*L of extracted DNA. To standardize the PCR protocol, suspensions of HeLa and SiHa cells naturally infected with HPV-18 and HPV-16, respectively, were used as positive controls. Distilled water was used as the negative control. The thermal cycling conditions were denaturation of DNA template and activation of DNA polymerase at 94°C for 7 minutes, 37 cycles at 94°C for 30 seconds, at 45°C for 45 seconds, and at 72°C for 1 minute, and a final extension at 72°C for 10 minutes. PCR products were run on a 10% nondenaturing polyacrylamide gel, followed by silver staining as described by Sanguinetti et al. [16], and classified as HPV positive if a band of 150 bp was identified when compared to a 100 bp ladder marker.

A data bank was generated and analyzed in GraphPad Prism, version 4.00. Prevalence ratios (PRs) for variables of interest with 95% confidence intervals (95% CI) were calculated. A two-tailed *P*-value of <.05 was regarded as significant; for statistical analysis, Student's *t*-test, Mann-Whitney test, and Fischer's Exact test were employed.

## 3. Results

Table 1 shows the characteristics of participants by HPV and HIV status. HIV-positive pregnant women had a significantly higher proportion of HPV infection than HIV-negative pregnant women (79.5% versus 58.5%; *P* < .05). HIV-positive serostatus was a risk factor for HPV positivity (PR 1.36; 95% IC 1.04–1.8; *P* = .03).

CD4 T cell count above 500 cells/mm^3^ and HIV RNA viral load below 1000 copies/mL were identified in 45% and 50% of HIV-positive pregnant women, respectively. All HIV-infected pregnant women with less than 200 CD4 T cells/mm^3^ or more than 10 000 HIV RNA copies/mL were HPV DNA positive. In the global context, there was significant association between HPV positivity and viral load (*P* < .05), but there was no clear association between HPV positivity and CD4 T cell count (*P* = .067), as showed in Table 1. Other characteristics were not significantly different according to HPV status.

## 4. Discussion

The prevalence of HPV infection detected in the present study was high among both HIV-positive and −negative pregnant women (79.5% and 58.5%, resp.), with a significant difference between the two groups. Few studies have been conducted on HIV-positive pregnant women. Minkoff et al. [12] suggested that HPV infection was not affected by pregnancy status among HIV-infected women. Our results are similar to findings reported in other studies [4, 6, 17], which identified higher HPV DNA positivity among HIV-infected nonpregnant women.

Higher viral load levels were associated with HPV positivity in the present study, which is in accordance with earlier studies [4, 8, 11]. Although we have not observed significant association between CD4 T cell count and HPV positivity, probably due to our limited sample of cases, a trend to this association is already possible to identify.

HPV DNA positivity was also high among HIV-uninfected pregnant women. Oliveira et al. [18] found similar HPV positivity rates in Brazilian HIV-negative women. This finding could also be due to the high percentage of young women, aged less than 25 years in this group, since age is one of the main risk factors for HPV infection [2]. In the same region of this study, Santos [19] found almost 52% of HPV positivity in pregnant teenagers. One weakness of our method might be that due to the absence of a nonpregnant women group, the possible immunosuppressive effect of gestation increasing the HPV rates cannot be assumed based on our study. A comparison with nonpregnant women could help elucidate this finding. In this context, another study conducted at our institution detected a 30% rate of HPV infection in HIV-uninfected women [20]. 

Even though the limited sample of cases and the target population, based on a tertiary health service, may restrict the generalization of our data, important results have already been demonstrated in the present study. According to our data, there is a significant association between HPV DNA positivity and HIV serostatus. Higher HIV viral load seems to be a major factor associated with HPV-HIV interaction. Short- and long-term active surveillance is needed to issue a definitive statement regarding the clinical significance of this finding during pregnancy for HIV infected women.

## Figures and Tables

**Table 1:** Characteristics of participants by HPV and HIV serostatus.

Characteristics	HPV-positive	HPV-negative	*P*-value
	*n* = 66	*n* = 31	
Number and percentage of HIV-positive	35/66 (53.03%)	9/31 (29.03%)	<**.05**
Number and percentage of HIV-negative	31/66 (46.97%)	22/31 (70.97%)	
Mean age (SD) of HIV-positive	27.2 (6.19)	28.2 (4.81)	.65
Mean age (SD) of HIV-negative	23.97 (6.16)	23.23 (5.67)	.86
Mean CD4 T cell count (SD) in HIV-positive (cells/mm^3^)	419.2 (240.2)	582.4 (191.8)	.067
Mean (SD) viral load in HIV-positive (copies/mL)	31,628 (86,848)	1,610 (3,151)	<**.05**
Mean number (SD) of previous pregnancies in HIV-positive	3.9 (2.2)	3.1 (1.3)	.38
Mean number (SD) of previous pregnancies in HIV-negative	2.4 (1.7)	1.9 (1.2)	.21
Mean age (SD) at first sexual intercourse in HIV-positive	15.8 (2.55)	15.7 (2.12)	.92
Mean age (SD) at first sexual intercourse in HIV-negative	16.2 (2.46)	16.6 (3.47)	.78

Marital status in HIV-positive			
* *Married/cohabiting	24	9	.08
* *Single/divorced/widowed	11	0	

Marital status in HIV-negative			
* *Married/cohabiting	20	16	.57
* *Single/divorced/widowed	11	6	

Lifetime number of sexual partners in HIV-positive			
* * ≤5	22	8	.46
* * >5	12	2	

Lifetime number of sexual partners in HIV-negative			
* * ≤5	29	19	.63
* * >5	2	3	
Number and percentage of previous STD in HIV-positive	14/35 (40%)	1/9 (11.1%)	.13
Percentage of previous STD in HIV-negative	4/31 (12.9%)	0/22 (0%)	.13
Percentage of past/current smokers in HIV-positive	13/35 (37.1%)	1/9 (11.1%)	.23
Percentage of past/current smokers in HIV-negative	10/31 (32.3%)	4/22 (18.2%)	.35

SD: Standard deviation;

STD: Sexually transmitted diseases.
